# Prosthetic valve endocarditis caused by multidrug-resistant *Candida albicans* in a patient with myelodysplasia syndrome: A case report and literature review

**DOI:** 10.18502/cmm.4.3.171

**Published:** 2018-09

**Authors:** Firoozeh Kermani, Tahereh Shokohi, Mahdi Abastabar, Lotfollah Davoodi, Shervin Ziabakhsh Tabari, Rozita Jalalian, Shirin Mehdipour, Roghayeh Mirzakhani

**Affiliations:** 1 Student Research Committee, School of Medicine, Mazandaran University of Medical Sciences, Sari, Iran; 2 Department of Medical Mycology, School of Medicine, Mazandaran University of Medical Sciences, Sari, Iran; 3 Invasive Fungi Research Centre, Mazandaran University of Medical Sciences, Sari, Iran; 4 Antimicrobial Resistance Research Center, Department of Infectious Diseases, Mazandaran University of Medical Sciences, Sari, Iran; 5 Department of Cardiac Surgery, Cardiovascular Research Center of Mazandaran Heart Center, Mazandaran University of Medical Sciences, Sari, Iran; 6 Department of Cardiology, Mazandaran University of Medical Sciences, Sari, Iran; 7 Mazandaran Heart Center, Mazandaran University of Medical Sciences, Sari, Iran

**Keywords:** Amphotericin B, Antifungal resistant, Azoles, *Candida *endocarditis, Multi-drug resistant, Myelodysplasia syndrome, Prosthetic valve replacement

## Abstract

**Background and Purpose:**

*Candida* endocarditis is an infrequent disease with a high mortality rate, which commonly occurs in immunosuppressed patients with cardiac valve replacement. We reported a 70-year-old woman diagnosed with *Candida* prosthetic valve endocarditis (PVE). This study also involved a review of all published cases of *Candida *PVE from 1970.

**Case report:**

Herein, we reported a 70-year-old woman with the history of severe mitral stenosis and myelodysplasia syndrome. She underwent mitral valve replacement for two times. The blood cultures were positive, and phenotypic identification of the isolates at the species level was performed based on microscopic and macroscopic characteristics. In the second prosthetic valve replacement, huge fungal white and creamy vegetation was observed which was identified as *Candida albicans *based on the conventional and molecular methods. Despite the administration of antifungal treatments, the patient passed away probably due to the multidrug-resistant *Candida* PVE.

**Conclusion:**

As PVE is a late consequence of prosthetic valve replacement, extended follow-up visits, early diagnosis, repeating valve replacement surgeries, and timely selective antifungal treatments are warranted.

## Introduction

*Candida* endocarditis is an infrequent disease with a high mortality rate, ranging within 23-46%. This disease is commonly reported in patients with cardiac valve replacement [1, 2]. The potentiality of *Candida* species, mainly *Candida albicans,* to form biofilms on native cardiac tissue and prosthetic valve causing increasing resistance to antifungal treatment, has a great impact on patient outcomes [3]. As *Candida* species have various degrees of susceptibility to currently available antifungals, it is very important to identify the causative agents and prescribe the effective treatment [3]. 

The major risk factors for this condition include indwelling central venous catheters, long-term broad-spectrum antibacterial therapy, and previous heart surgery. Other risk factors, such as long-term usage of corticosteroids, cytotoxic drug consumption, intravenous drug abuse, and immunosuppression, are implicated as the causes of increasing the incidence of fungal endocarditis [4, 5]. In recent years, the emergence of drug-resistant *Candida* species has complicated the treatment of different diseases and adoption of the proper medication regimen.

Herein, we reported a case of *Candida* prosthetic valve endocarditis (PVE) caused by multi-azole and amphotericin B resistance in a patient with myelodysplasia syndrome (MDS) for the first time in Iran. We also briefly reviewed the reports of PVE caused by *Candida *species in patients with different types of cancers.

## Case report

A 70-year-old woman with a severe headache, vertigo, fever, and arrhythmia, suspected with Parkinson's disease was admitted to Mazandaran Heart Center, Sari, north of Iran, in 2017. She had a history of symptomatic sever mitral stenosis probably due to rheumatic heart disease in her childhood for which she underwent mitral commissurotomy when she was 25 years old. After her first surgery, she administered penicillin G benzathine 1.2 million units IM once a month up to her last admission. She was also subjected to echocardiography every 6 months.

In 2010, the patient suffered from persistent fever, which was unresponsive to antibiotics for 2 weeks. Echocardiography revealed infective endocarditis; however, no microbial strain was isolated from blood culture. After treatment, symptoms reduced; nonetheless, a few days after discharging from the hospital, her blood cell profile was deteriorated gradually. The diagnosis of MDS was eventually confirmed after performing bone marrow aspiration for three times. Thereafter, the patient was subjected to androgen therapy with danazol (10 mg/kg b.w./day), and Prednisolone (1 mg/kg b.w./day). When the white blood cell count became normal, danazol was discontinued; however, the consumption of prednisolone (5 mg/day) was continued. During the long-term use of prednisolone, she was afflicted with steroid-induced diabetes and oral lichen planus due to the impairment of immune system. 

On October 2015, due to the deterioration of patient's general condition, she was transferred to Tehran Heart Center for further evaluation. Transesophageal echocardiography (TEE) revealed severe mitral valve (MV) stenoses; as a result, she was subjected to percutaneous transvenous mitral commissurotomy.

On May 2016, the patient was admitted to hospital due to persistent fever and general weakness, and was detected with MV regurgitation. She was prescribed vancomycin (20 mg/kg), gentamicin (1 mg/kg), and ciprofloxacin (10 mg/kg). She had a fever of up to 40°C that was unresponsive to antibiotics and persisted after a week. Imipenem was replaced with ciprofloxacin and continued for 6 weeks. The TEE showed mobile MV vegetation that involved more than 3/4 of the valves. Therefore, the patient underwent MV replacement with a biological (porcine) prosthetic valve.

Following the use of broad-spectrum antibiotics, she was diagnosed with *Candida* onychomycosis and recurrent *Candida *vulvovaginitis, and therefore prescribed fluconazole (150 mg) for 3 weeks and caspofungin (intravenous [IV]; 50 mg three times a day) for 6 weeks, coupled with vancomycin and gentamicin. 

On February 2017, she presented flu-like symptoms, such as fever and chills, dizziness, severe headache, and heart arrhythmias for 5 days prior to hospital admission. She was admitted to the Cardiac Care Unit of Mazandaran Heart Center in Sari and prescribed ceftizoxime (IV, 500 mg). Echocardiography revealed a large vegetation on the MV annulus. 

Two consecutive blood samples were obtained from the patient and inoculated into biphasic brain heart infusion medium. After one week of incubation at 37°C, the blood cultures were positive. The yeast isolate was presumptively identified as *C. albicans* using conventional methods including chlamydospore production test, germ tube test, and appearance on CHROMagar *Candida*. 

On March 2017, the patient’s condition deteriorated. The blood cultures were negative. She underwent the second prosthetic valve replacement. The surgery revealed the formation of huge fungal white and creamy vegetation and abscess overall the prosthetic valve. The explanted valve and vegetation were sent to laboratory for further evaluations. Direct examination (KOH 20%) and calcofluor white staining of the sample showed lots of budding yeast cells. 

To identify the species, the yeast colonies were yielded on Sabouraud dextrose agar medium after a 24-hour incubation at 27ºC. The isolated yeast was identified as *Candida albicans* using the conventional methods. The diagnosis was confirmed by polymerase chain reaction (PCR) assay using the two universal primers, namely ITS1 and ITS4 [6]. The amplicons were sequenced and compared with the GenBank database; then, they were submitted to GenBank and received accession number MG763751.

Amphotericin B, deoxycolate (1 mg/kg/day), caspofungin (70 mg/kg on the first day and 50 mg/kg in the next days), and voriconazole (6 mg/kg bid on the first day and 4 mg/kg bid in the next days) together with broad-spectrum antibiotics, including vancomycin (20 mg/kg bid) and gentamicin (1 mg/kg bid) were administered. However, 48 h later, the patient presented with dyspnea, decreased consciousness, and decreased blood cells, resulting in a coma. The patient passed away due to sepsis probably related to the candidemia and *Candida* PVE with antifungal-resistant *Candida* *albicans.*

In vitro antifungal susceptibility testing of *Candida* *albicans* isolate was carried out based on the clinical and laboratory standards institute (CLSI) M27-A3 [7] and M27-S4 guidelines [8]. Based on the breakpoint, the isolate was resistant to voriconazole (16 μg/ml), itraconazole (16 μg/ml), fluconazole (64 μg/ml), posaconazole (16 μg/ml), and amphotericin B (4 μg/ml). Furthermore, it was susceptible to anidulafungin (0.008 μg/ml) and micafungin (0.008 μg/ml), and intermediate to caspofungin (0.5 μg/ml).

***Ethical considerations***

The study protocol was approved by the Ethics Committee of Mazandaran University of Medical Sciences, Sari, Iran.

## Discussion

Over the past few years, there has been an increasing number of reports on fungal endocarditis [9] showing the high morbidity and mortality rate of this condition, ranging within 30-80% [10]. Although *Candida* endocarditis accounts for about 1-2% of infective endocarditis, it can be very fatal. This medical condition is usually diagnosed postmortem because of its nonspecific clinical symptoms. *C. albicans,* followed by *C. parapsilosis,* are the common *Candida *species causing endocarditis [9].

According to the Duke criteria, PVE is classified as ''early'' when it happens within 60 days of valve replacement and ''late'' when occuring more than 60 days post-replacement [11, 12]. In this report, our case was classified as a late *Candida* endocarditis because symptoms manifested 300 days after the first valve replacement. 

In our patient, the major predisposing factors included a congenital heart disease, malignancy such as myelodysplastic syndromes (formerly described as pre-leukemia or smoldering acute leukemia), long-term use of corticosteroids for MDS treatment, prosthetic cardiac valves, and long-term broad-spectrum antibiotic therapy after cardiac surgery.

To review the cases of prosthetic valve endocarditis caused by *Candida* species in patients with different types of cancers, a search was performed on the English articles published from 1970 onward in two databases, namely Google Scholar and PubMed. The key words and medical subject headings used for the search were as follows: “*Candida* endocarditis”, “Prosthetic valve endocarditis”, and “Cancer”. Table 1 summarizes the demographic features, risk factors, treatment strategies, and outcomes in reported cases of prosthetic valve endocarditis caused by *Candida* species in patients with different types of cancers.

**Table 1:** Demographic features, risk factors, treatment, and outcome in reported cases of prosthetic valve endocarditis caused by *Candida* species with different types of cancers

**Reference**	**Country**	**Age/** **gender**	**Type of cancer**	**Treatment**	**Diagnosis method**	**Causative agent**	**Risk factors**	**Observation**	**Outcome**
Ihde, 1978 [13]	USA	<20/ NS	Lymphoma, carcinoma of the cervix	Corticosteroids, antibiotics	B/C(+),CSF/C (+), Microscopic examination	*C. albicans*	Chemotherapy, central venous catheter, corticosteroids	Abscesses in the left ventricular myocardium	Died
Maeno, 1990 [14]	Japan	83/ NS	Pancreatic cancer	Anti-fungal agents	B/C (+), mannan antigenemia, D-arabinitol creatinine ratio, Echo	*C. albicans*	NS	Vegetation at the aortic valve	Died
Johnston, 1991 [15]	USA	31/M	Testicular carcinoma	5-FC+AMB, surgery	B/C (+), TEE	*C. albicans*	recurrent embryonal cell testicular carcinoma	Mitral valve vegetation	Died
Hamada, 1996 [16]	Japan	63/M	Gastric cancer	Anti-fungal agents, surgery	B/C(+)	*C. albicans*	liver abscess	Aortic and tricuspid regurgitation	Survived
		65/M	Bile duct cancer	Anti-fungal agents, catecholamine, digoxin, surgery	B/C(+)	*C. albicans*	NS	Aortic regurgitation	Died
Inoue, 1998 [17]	Japan	57/M	Gastric cancer	FLC, surgery	NS	*C. parapsilosis*	Chemotherapy, central venous catheter	Cardiac valve vegetation	Survived
Ariffin, 1999 [18]	Malaysia	<1/NS	ALL	AMB , FLC	Echo	*C. albicans*	Immunodeficiency	NS	NS*
Jagernauth, 2007 [19]	UK	54/M	Carcinoid disease	Antifungal drug, Surgery	NS	*Candida *spp	Surgery for pulmonary and tricuspid valve replacement	Vegetation	Survived
Ozkiraz, 2007 [20]	Turkey	<1/ NS	AML	FLC, AMB , surgery	Echo, B/C(+), pathological examination	*C. albicans*	NS	Vegetation at the outlet of the right ventricle	Died
Block, 2009 [21]	Australia	64/F	Carcinoma of the lung	FLC, 5FC thrombolytic therapy, surgery	B/C (+), U/C(+), TEE	*C. albicans*	NS	Fungal ball	Survived
Chopra, 2010 [22]	USA	74/F	Cardiomyopathy	MFG, surgery	B/C, U/C(+), TEE	*C. kefyr*	Diabetes type II	Mitral valve vegetation	Survived
Reyes, 2015 [23]	Peru	36/F	Ovarian cancer	AFG, VRC , FLC, antibiotics, surgery	B/C(+), TEE	*C. parapsilosis*	Chemotherapy	Vegetation in the aortic valve	Survived
Present case	Iran	70/F	MDS	AMB deoxycolate, CAS, VRC	B/C(+), TEE	*C. albicans*	Spectrum antibiotic therapy, diabetes	Mitral valve vegetation	Died

Ns: not specified, TTE: transthoracic echocardiography, TEE: transesophageal echocardiography, ALL: acute lymphoblastic leukemia, MDS: myelodysplasia syndrome, Echo: echocardiography, FLC: fluconazole, AMB: amphotericin B, AFG: anidulafungin, CAS: caspofungin, VRC: voriconazole, MFG: micafungin, 5FC: flucytosine, B/C: blood culture, U/C: urine culture, CSF: cerebrospinal fluid culture

The main factor that made treatment unresponsive was associated with multi-azole and amphotericin B-resistant *Candia *endocarditis. Multiple risk factors in our patient might be more likely to cause fungal endocarditis and have a fatal outcome. The combination of risk factors, clinical features, and echocardiography findings may help the clinical diagnosis of endocarditis. The TEE is a modality of choice with high sensitivity (87-100%) and specificity (83-94%) in the initial evaluation of the patients with high risk for infective endocarditis [24].

The blood culture as a traditional gold standard for the detection of candidemia has some limitations, including low sensitivity and high turnaround time [25]. Blood culture was positive in the first valve replacement surgery, but it was negative in the second one, while TEE showed a large vegetation on the mitral valve leading to endocarditis. The accurate diagnosis of *Candida *endocarditis is a challenge since its symptoms are very similar to those of bacterial infections. Negative blood culture results can lead to delayed anti-fungal therapy that contributes to an increased risk of hospital mortality. This explains why most of *Candida* PVE cases are diagnosed on autopsy or very late [2]. 

*Candida* species have wide ranges of virulence factors, such as adhesion, invasion, and biofilm formation on medical devices, such as biotic or artificial cardiac valves. They can have detrimental effects on patient's life, because they lead to the failure of the prostheses and result in the persistent presence of organism in bloodstream as a source of fungemia episodes [26].

Azole-resistant *Candida* species is regarded as a considerable problem for patients undergoing long-term fluconazole treatment. There are only four drug classes for *Candida* infections, including azoles, polyenes, echinocandins, and pyrimidine analogue of cytosine (5-fluorocytosine). Multidrug-resistance is defined as the non-susceptibility of the isolate to ≥ 1 agent in ≥ 2 antimicrobial categories [27]. 

In this case, we reported *C. albicans* that was resistant to multi-azoles and amphotericin B. 

Hematologic disorders, such as MDS, are reported as risk factors for invasive *Candida* infections [28]. Long-term use of corticosteroids for the treatment of MDS and prolonged use of broad-spectrum antibiotics (e.g., penicillin over 35 years) can severely lead to the conditions predisposing a patient to candidemia and *Candida* endocarditis. Candidemia after heart valve replacement is a powerful predictive factor for late PVE, even more than one year, after the first episode of infection. Therefore, patients should be checked with an extended follow-up.

*Candida *endocarditis is an uncommon but devastating infection that affects the elderly with a weakened immune system as a late consequence of prosthetic valve replacement. The extended follow-up visits, early diagnosis, repeating valve replacement surgeries, and timely selective antifungal treatments are warranted.

## Conclusion

This report highlighted that *Candida* PVE can occur as a late consequence of valve replacement in elderly patient with suppressed immune system. The extended follow-up visits, early diagnosis, repeating valve replacement surgeries, and timely selective antifungal treatments are warranted.

## References

[1] Abgueguen P, Gouello JP, Pichard E, Chabasse D, Donal E, Alquier P (2002). Candida endocarditis: retrospective study in 12 patients. Rev Med Interne.

[2] Shokohi T, Nouraei SM, Afsarian MH, Najafi N, Mehdipour S (2014). Fungal prosthetic valve endocarditis by Candida parapsilosis: a case report. Jundishapur J Microbiol.

[3] Rubinstein E, Lang R (1995). Fungal endocarditis. Eur Heart J.

[4] Yuan SM (2016). Fungal endocarditis. Braz J Cardiovasc Surg.

[5] Fesharaki SH, Haghani I, Mousavi B, Kargar ML, Boroumand M, Anvari MS (2013). Endocarditis due to a co-infection of Candida albicans and Candida tropicalis in a drug abuser. J Med Microbiol.

[6] White TJ, Bruns T, Lee SJ, Taylor JL (1990). Amplification and direct sequencing of fungal ribosomal RNA genes for phylogenetics. PCR Protoc Guide Methods Appl.

[7] Wayne PA (2008). Clinical and laboratory standards institute. Reference method for broth dilution antifungal susceptibility testing of yeasts; approved standard. CLSI Document M27-A3.

[8] Pfaller MA, Diekema DJ (2012). Progress in antifungal susceptibility testing of Candida spp using clinical and laboratory standards institute broth microdilution methods, 2010-2012. J Clin Microbiol.

[9] Azhar A (2012). Successful management of fungal pericarditis and endocarditis in a neonate: a case report. J Saudi Heart Assoc.

[10] Ellis ME, Al-Abdely H, Sandridge A, Greer W, Ventura W (2001). Fungal endocarditis: evidence in the world literature, 1965-1995. Clin Infect Dis.

[11] Nguyen MH, Nguyen ML, Yu VL, McMahon D, Keys TF, Amidi M (1996). Candida prosthetic valve endocarditis: prospective study of six cases and review of the literature. Clin Infect Dis.

[12] Durack DT, Lukes AS, Bright DK (1994). New criteria for diagnosis of infective endocarditis: utilization of specific echocardiographic findings Duke Endocarditis Service. Am J Med.

[13] Ihde DC, Roberts WC, Marr KC, Brereton HD, McGuire WP, Levine AS (1978). Cardiac candidiasis in cancer patients. Cancer.

[14] Maeno K, Hirota S, Kubota K, Takata S, Ikeda T, Kobayachi K (1990). A case of Candida endocarditis with consecutive measurement of serum mannan and D-arabinitol concentrations. Kokyu To Junkan.

[15] Johnston PG, Lee J, Domanski M, Dressler F, Tucker E, Rothenberg M (1991). Late recurrent Candida endocarditis. Chest.

[16] Hamada Y, Yamada M, Furuno T, Fujii C, Matsumura Y, Yabe T (1996). Two cases of Candida endocarditis associated with abdominal disease. Nihon Ronen Igakkai Zasshi.

[17] Inoue Y, Yozu R, Ueda T, Kawada S (1998). A case report of Candida parapsilosis endocarditis. J Heart Valve Dis.

[18] Ariffin H, Ariffin W, Tharam S, Omar A, de Bruyne J, Lin HP (1999). Successful treatment of Candidaalbicans endocarditis in a child with leukemia--a case report and review of the literature. Singapore Med J.

[19] Jagernauth S, Patel A, Baig K, De Souza A (2007). Fungal endocarditis of the eustachian valve in carcinoid heart disease: a case report. J Heart Valve Dis.

[20] Ozkiraz S, Tarcan A, Gokmen Z, Gurakan B, Bilezikci B, Ozbek N (2007). Invasive Candidaalbicans infection mimicking leukemia in a neonate. J Matern Fetal Neonatal Med.

[21] Block AA, Thursky KA, Worth LJ, Slavin MA (2009). Thrombolytic therapy for management of complicated catheter-related Candidaalbicans thrombophlebitis. Intern Med J.

[22] Chopra T, Bhargava A, Kumar S, Chopra A, Dhar S, Afonso L (2010). Candida kefyr endocarditis in a patient with hypertrophic obstructive cardiomyopathy. Am J Med Sci.

[23] Reyes HA, Carbajal WH, Valdez LM, Lozada C (2015). Successful medical treatment of infective endocarditis caused by Candida parapsilosis in an immunocompromised patient. BMJ Case Rep.

[24] Rubinstein E, Lang R (1995). Fungal endocarditis. Eur Heart J.

[25] Arvanitis M, Anagnostou T, Fuchs BB, Caliendo AM, Mylonakis E (2014). Molecular and non-molecular diagnostic methods for invasive fungal infections. Clin Microbiol Rev.

[26] Ramage G, Saville SP, Thomas DP, Lopez-Ribot JL (2005). Candida biofilms: an update. Eukaryot Cell.

[27] Arendrup MC, Patterson TF (2017). Multidrug-resistant Candida: epidemiology, molecular mechanisms, and treatment. J Infect Dis.

[28] Boktour MR, Kontoyiannis DP, Hanna HA, Hachem RY, Girgawy E, Bodey GP (2004). Multiple-species candidemia in patients with cancer. Cancer.

